# Seasonable Items

**Published:** 1879-10

**Authors:** 


Very many are made sick in the Fall, on their return from their summer diversions, by failing to kindle fires promptly morning and evening when the weather becomes chilly. Women especially are found going about the house shivering in their shawls ; this shows that the skin is cold, the pores are contracted ; this drives the blood upon the internal organs in excessive quantity ; if on the lungs, there is Pneumonia, called inflammation of the lungs ; if on the windpipe, it causes Bronchitis ; if on the nose, it is Catarrh ; if on the liver, there is biliousness, with its varying symptoms of want of appetite, headache, cold feet, constipation, sick stomach, and a general miserableness all over, with low spirits, bad temper, and all its unfortunate consequences on the whole household ; and the victims begin to wonder why going to the country did them no good, not knowing that laziness is at the very foundation of it all. Fires are not kindled because it is troublesome ; it makes dust, and "it will last only for a day or two."

Whenever in September the morning is cool enough to attract attention unpleasantly, and make one think of a blazing fire on the hearth and its comfort, that fire ought to be kindled within half an hour, and repeated before sunrise and before sunset, whether the weather is warm or cool, until steady fires are needed. Have such a fire, at least in one room in the house, to be resorted to or not, according to the feelings of the individual members.

These fires are needed quite as much, although they may make the room too warm for comfort ; then let the inner doors be open—the result will be that the miasm which causes fever and ague about that time of year is rarified and carried up the chimney, or left near the ceiling where it can do no harm. Any family acting thus in aguish localities will be entirely exempt if they take breakfast before they go out of doors, and take supper at sundown in the same warm room.

Whenever the thermometer is below seventy degrees out of doors in spring or autumn, there ought to be an open fire in at least one room in the house. Money will be saved, and a vast amount of trouble and sickness every fall can be prevented in this way.

